# Editorial April 2021

**DOI:** 10.1093/braincomms/fcab059

**Published:** 2021-04-27

**Authors:** Tara L Spires-Jones

**Affiliations:** UK Dementia Research Institute and Centre for Discovery Brain Sciences, the University of Edinburgh, 1 George Square, Edinburgh EH8 9JZ, UK

Welcome to Volume 3, Issue 2, of *Brain Communications*! The volume of our published papers has steadily increased, despite the difficulties, many researchers have faced during 2020. Thus, in 2021, we are moving to having more issues per year. Since Volume 3, Issue 1 opened in January, we have continued to have growing submissions and have had the privilege to publish fascinating and important work. A couple of original research articles to highlight are: Kalyanaraman and team’s discovery of a pentanucleotide expansion in intron 4 of the *SAMD12* gene as a causal mutation for autosomal dominant cortical tremor, myoclonus and epilepsy in a unique ethnic group in South India^1^; and Maeda and teams^2^ report of down-regulation of microglia P2Y12 receptor associated with tau rather than amyloid-β pathologies from an early stage in models of neurodegeneration. We are also continuing to promote rigour and transparency in translational neuroscience, including publishing a protocol for a phase I clinical trial of foetal cell transplantation in Huntington’s disease.^3^

In our last issue, we received a letter to the editor^4^ discussing a review of nodding syndrome published last year and a response from the original authors.^5^ This stimulating discussion of the ongoing literature around causes and prevention of nodding syndrome highlights the growing community of *Brain Communications* authors, reviewers and readers. I hope this community will continue to grow. Please join the discussions online with our @BrainComms twitter account, or do get in touch if you have any ideas for letters to the editor, scientific commentaries on our papers, or field potential articles about advances in translational neuroscience and career development of early career researchers in the field. At the first *Brain* conference in March, I got the chance to briefly introduce our journal to the >700 participants before hearing some inspiring scientific talks, teaching talks and perspectives on the history of the journal *Brain* by Alastair Compston. Hopefully many attendees will be inspired to engage with us after hearing these talks and seeing the excellent posters at the conference.

Finally, I would like to welcome Prof Masud Husain’s new team at our sister journal *Brain* who have officially started their tenure running the journal. They have implemented some formatting changes which you will soon see in our papers as we will keep the style consistent in the family.

The cover image for this issue comes from Balestrini et al.^6^ who have observed increased face and brain asymmetry in focal epilepsies.

## Competing interests

The authors report no competing interests.
